# Immunological mechanisms involved in macrophage activation and polarization in schistosomiasis

**DOI:** 10.1017/S0031182023000021

**Published:** 2023-04

**Authors:** Irlla Correia Lima Licá, Gleycka Cristine Carvalho Gomes Frazão, Ranielly Araujo Nogueira, Maria Gabriela Sampaio Lira, Vitor Augusto Ferreira dos Santos, João Gustavo Mendes Rodrigues, Guilherme Silva Miranda, Rafael Cardoso Carvalho, Lucilene Amorim Silva, Rosane Nassar Meireles Guerra, Flávia Raquel Fernandes Nascimento

**Affiliations:** 1 Graduate Program in Health Sciences, Center for Biological and Health Sciences, Federal University of Maranhão, São Luís, MA, Brazil; 2 Laboratory of Immunophysiology, Center for Biological and Health Sciences, Federal University of Maranhão, São Luís, MA, Brazil; 3 Department of Parasitology, Institute of Biological Sciences, Federal University of Minas Gerais, Belo Horizonte, Minas Gerais, Brazil; 4 Department of Biology, Federal Institute of Education, Science and Technology of Maranhão, São Raimundo das Mangabeiras, Brazil; 5 Department of Pathology, Center for Biological and Health Sciences, Federal University of Maranhão, São Luís, MA, Brazil

**Keywords:** Granuloma, M1, M2, phagocytes, schistosomiasis

## Abstract

Human schistosomiasis is caused by helminths of the genus *Schistosoma*. Macrophages play a crucial role in the immune regulation of this disease. These cells acquire different phenotypes depending on the type of stimulus they receive. M1 macrophages can be ‘classically activated’ and can display a proinflammatory phenotype. M2 or ‘alternatively activated’ macrophages are considered anti-inflammatory cells. Despite the relevance of macrophages in controlling infections, the role of the functional types of these cells in schistosomiasis is unclear. This review highlights different molecules and/or macrophage activation and polarization pathways during *Schistosoma mansoni* and *Schistosoma japonicum* infection. This review is based on original and review articles obtained through searches in major databases, including Scopus, Google Scholar, ACS, PubMed, Wiley, Scielo, Web of Science, LILACS and ScienceDirect. Our findings emphasize the importance of *S. mansoni* and *S. japonicum* antigens in macrophage polarization, as they exert immunomodulatory effects in different stages of the disease and are therefore important as therapeutic targets for schistosomiasis and in vaccine development. A combination of different antigens can provide greater protection, as it possibly stimulates an adequate immune response for an M1 or M2 profile and leads to host resistance; however, this warrants *in vitro* and *in vivo* studies.

## Graphical abstract

**Figure:**
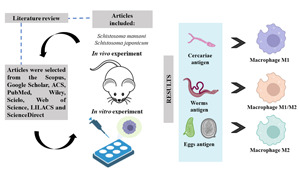

## Introduction

Human schistosomiasis is a neglected parasitic disease with great relevance to public health. Worldwide, it is estimated that approximately 230–250 people are infected and 700–800 million live in areas that are at risk of infection, mainly in countries located in South America, Asia and Africa (Steinmann *et al*., 2006; Colley *et al*., 2014; McManus *et al*., 2018; Wei *et al*., 2018; WHO, 2020). In addition, approximately 200 000–280 000 deaths occur each year due to schistosomiasis and its complications (LoVerde, 2019). The high prevalence of schistosomiasis is mainly related to people living in extreme poverty and poor sanitation, which represent a serious risk to human health (Ismail *et al*., 2014; Bajiro *et al*., 2017; Verjee, 2019).

The infection is caused by helminths of the genus *Schistosoma* (Colley *et al*., 2014; Stingl and Stingl, 2017; WHO, 2020), belonging to the class Trematoda and phylum Platyhelminthes. The main aetiologic agents of this disease, in terms of clinical relevance, are *Schistosoma japonicum*, *Schistosoma mansoni* and *Schistosoma haematobium* (WHO, 2020). In this review, we focus only on *S. mansoni* and *S. japonicum*, as they are the main species associated with hepatic and intestinal schistosomiasis (Wilson *et al*., 2007; Chen *et al*., 2013; McManus *et al*., 2018).

There are 2 distinct phases of clinical progression of intestinal schistosomiasis: the acute and the chronic phases (Gobbi *et al*., 2020). During the early stages of acute phase of schistosomiasis (before parasite oviposition), there is a predominance of the T helper type 1 (Th1) immune response (Pearce *et al*., 1991; Hesse *et al*., 2001; Pearce and MacDonald, 2002; Colley and Secor, 2014). After schistosome oviposition, the immune response becomes strongly polarized to the Th2 profile, which is related to increasing production of interleukin-4 (IL-4), IL-5, IL-9 and IL-13 (Pearce and MacDonald, 2002; Burke *et al*., 2009). This immune environment is responsible for the formation of granulomas in tissues (Grzych *et al*., 1991; Brunet *et al*., 1997; Hoffmann *et al*., 2000). The granuloma has an important role for the host, because it contains the tissue damage caused by antigens secreted by the schistosome eggs (Hams *et al*., 2013; Schwartz and Fallon, 2018). In the chronic phase of schistosomiasis, there is an increase in the production of regulatory cells in the granuloma, which can modulate granulomatous inflammation, promoting a minimization of the disease severity (Hesse *et al*., 2004; Lundy and Lukacs, 2013). However, if this inflammatory reaction does not have an adequate modulation, the granulomas progressively may evolve into large areas of fibrosis, responsible for the main pathology of schistosomiasis (Hams *et al*., 2013; Schwartz and Fallon, 2018), including hepatosplenomegaly (Masi *et al*., 2020), portal hypertension (Grieco *et al*., 2016) and ascites (Fei-Yue *et al*., 2017).

Macrophages are cells of the innate immune system that play important roles in controlling infections (Shapouri-Moghaddam *et al*., 2018), as well as in tissue remodelling processes, both in ontogenesis and wound healing (Kloc *et al*., 2019). In the course of *S. mansoni* and *S. japonicum* infection, either at its initial stage or during the evolution to the chronic phase, macrophages participate in the immune regulation of the disease (Cortes-Selva *et al*., 2018; Ho *et al*., 2022).

Macrophages can acquire different phenotypes depending on the stimuli to which they are subjected to (Atri *et al*., 2018). These cells can be classified into M1 or ‘classically activated’ cells, with pro-inflammatory action, and M2 or ‘alternatively activated’ macrophages, which are considered anti-inflammatory cells (Mills, 2015; Ley, 2017; Locati *et al*., 2020). However, despite the relevance of macrophages in controlling infections, the participation of the functional types of these cells in acute and chronic schistosomiasis is still not well defined. Thus, this review discusses the different molecules and/or pathways of activation and polarization of macrophages during infection by *S. mansoni* and *S. japonicum*, leading to a better understanding of the role of these cells in the immunopathology of schistosomiasis. Based on this knowledge, we may help identify potential targets for the development of better treatment strategies to reduce the morbidity of this disease.

## Methods and criteria for literature selection

This literature review was performed using recognized databases including Scopus, Google Scholar, ACS, PubMed, Wiley, Scielo, Web of Science, LILACS and ScienceDirect and covered original and review articles published in English from 1966 to 2022. Articles involving *in vitro* and/or *in vivo* experiments were included and addressed the main immunological aspects of *S. mansoni* and *S. japonicum* infection related to macrophage polarization, activation and effector functions. To search for these articles, combinations of keywords were used, such as ‘Macrophage’, ‘*Schistosoma*’, ‘macrophage polarization and *Schistosoma*’, ‘Macrophage and Schistosoma’, ‘Macrophage and *Schistosoma mansoni*’, ‘Macrophage and *Schistosoma japonicum*’. Research involving coinfections was not included in this study.

## Immunopathology of the definitive host against infection by *S. mansoni* and *S. japonicum*

**Fig. 1.**
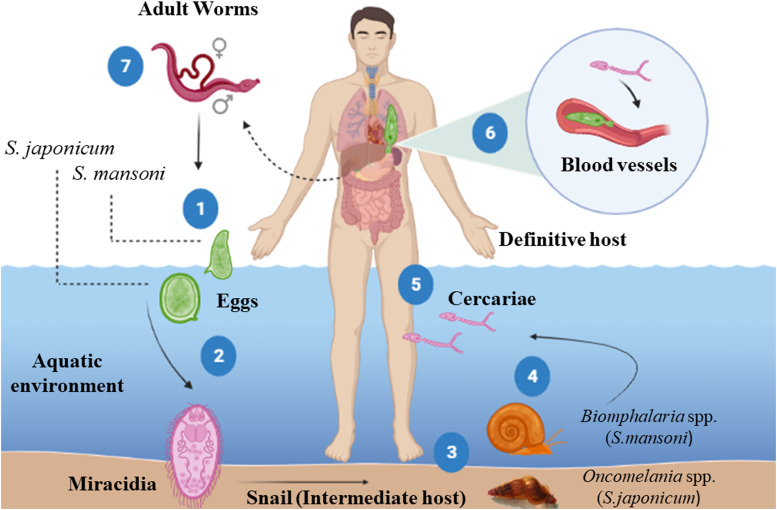
Life cycle of *Schistosoma mansoni* and *Schistosoma japonicum*. (1) The eggs shed in the feces of the definitive host release the miracidia when they come in contact with water (2), which penetrate in soft tissue the intermediate host snail (*Biomphalaria* spp./*Oncomelania* spp.). Inside the snail, the miracidia transform into mother sporocysts, which in turn produce daughter sporocysts by asexual reproduction. After around 30 days post-infection, cercariae emerge from the daughter sporocysts and are shedding by the snails in response to the light and heat (4). The cercariae penetrate the skin of the definitive host (5) and later transform into schistosomula. These larvae enter venous blood vessels and are passively carried to the lungs and heart (6). Upon reaching the hepatic portal system, schistosomula mature, become adult worms (male or female) and mate (7). The mated worms migrate to the lower mesenteric veins of the intestine, where the female sheds the eggs. Part of these eggs pass through the intestinal wall and are eliminated in the feces, starting the cycle again. However, some eggs are not eliminated and get trapped in several organs (mainly the liver and intestines), inducing a potent granulomatous inflammatory response, responsible for schistosomiasis pathology. *Source*: Created with BioRender.com.

**Fig. 2.**
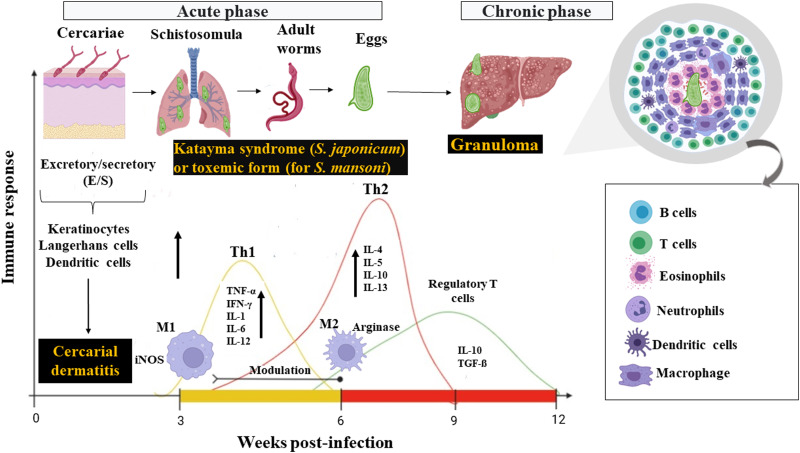
Different immune response profiles during *S. mansoni* and *S. japonicum* infection. *Source*: Created with BioRender.com.

Parasites of the genus *Schistosoma* have complex life cycles (Fig. 1), with generations of asexual reproducing larvae living in freshwater snails, the intermediate hosts (some species of the genus *Biomphalaria* for *S. mansoni* and the genus *Oncomelania* for *S. japonicum*) and another stage of sexual reproduction of adult worms in vertebrate hosts (definitive), including humans (McManus *et al*., 2018; Nelwan, 2019). Each stage of the parasite's life cycle (cercariae, schistosomulae, adult worms and eggs) within the definitive host triggers a series of immune responses, and consequently, clinical signs that can be harmful to humans (Molehin, 2020; Hambrook and Hanington, 2021; Masamba and Kappo, 2021). The interactions between the host immune system and the parasite can be divided into 2 phases (Fig. 2): acute phase (after and before parasite oviposition) and chronic phase (Gobbi *et al*., 2020).

The first clinical manifestations of the acute phase (cercarial dermatitis, oedema and pruritus) begin 48–72 h after cercariae penetrate the host's skin, and occurs mainly in individuals from endemic areas (frequently exposed to infection) (He *et al*., 1990, 2005; Khammo *et al*., 2002; Ingram *et al*., 2003; Lambertucci, 2010). The first innate immune barrier encountered by cercariae is the skin (Bartlett *et al*., 2000; Whitfield *et al*., 2003; He *et al*., 2005). This tissue is composed of keratinocytes, whose function is to secrete cytokines with antimicrobial functions (Roupé *et al*., 2010; Piipponen *et al*., 2020). Indeed, the keratinocytes are considered the first active cells in response to cercariae infection (Bourke *et al*., 2015). These cells rapidly respond to infections by secreting inflammatory cytokines [IL-6, IL-12, tumour necrosis factor-alpha (TNF-*α*) and IL-1*β*] to repair damaged tissue (Hogg *et al*., 2003*a*, 2003*b*). When penetrating the host's skin, cercariae also cause an increase in antigen-presenting cells in the innate immune system, such as Langerhans cells and dendritic cells (DCs), as shown in Fig. 2 (Angeli *et al*., 2001; Kumkate *et al*., 2007; Hambrook and Hanington, 2021), which contribute to a type 1 cellular immune response (He *et al*., 2005; Perona-Wright *et al*., 2006).

Initial immune responses are activated as a result of excretory/secretory (E/S) products released by the cercariae penetrating glands at the time of penetration into the host's skin (Salter *et al*., 2000; Jenkins *et al*., 2005*a*, 2005*b*; Curwen *et al*., 2006; Paveley *et al*., 2009). E/S products assist in the immunomodulatory function exerted by cercariae, as well as condition the remodelling of the extracellular matrix, facilitating its penetration into the skin (Janssen *et al*., 2016; Leontovyč *et al*., 2020). Liu *et al*. (2015) performed a proteomic analysis of products excreted by *S. japonicum* cercariae at the time of skin entry and identified a variety of E/S proteins, mainly proteases. Among the enzymes that allow this remodelling, the cercarial elastase of *S. mansoni* stands out, which is of great importance in the penetration of cercariae into the skin and can degrade a wide variety of macromolecules present in the human integument (Ingram *et al*., 2012; El-Faham *et al*., 2017).

Parasitic E/S products also promote the activation of prostaglandin E2 (PGE2) and prostaglandin D2-producing keratinocytes (Kaisar *et al*., 2018; Oyesola *et al*., 2021), which are molecules that induce the production of IL-10 *via* a cyclooxygenase 2-dependent pathway (Ramaswamy *et al*., 2000; Harizi *et al*., 2002; Xue *et al*., 2005). This type of response is responsible for modulating the immune response that favours parasite survival (Angeli *et al*., 2001; Hervé *et al*., 2003; De Oliveira Fraga *et al*., 2010). Abdel-Ghany *et al*. (2015) suggested that blocking PGE2 might provide partial protection in *S. mansoni*-infected mice. In addition, during the period when cercariae transform into schistosomules and migrate through the skin, PGE2 acts as a potent vasodilator, helping the passage of these larval forms into circulation (Ruzicka and Printz, 1984).

After penetrating the host's skin, cercariae undergo morphological and biochemical changes, transforming into juvenile forms, known as schistosomula, that reach blood vessels (Brink *et al*., 1977; Wilson, 1987; Curwen and Wilson, 2003). In the bloodstream, the schistosomula is passively transported to the lungs and heart until they finally reach the hepatic portal system, where they develop into adult male or female worms (Miller and Wilson, 1978; Wheater and Wilson, 1979; Nation *et al*., 2020) (Fig. 1). In this phase before the parasite's oviposition (early stages of acute phase), the host produces a predominantly type 1 immune response, which reaches greater activation between the 3rd and 5th weeks after exposure to cercariae (Dunne and Cooke, 2005; Gryseels *et al*., 2006). This response is characterized by high production of pro-inflammatory cytokines, such as IL-1, IL-2, IL-6, IL-12, interferon-gamma (IFN-*γ*) and TNF-*α* (Fig. 2) (Grzych *et al*., 1991; Pearce *et al*., 1991; Egesa *et al*., 2018; Zheng *et al*., 2020). Coinciding with the migration and sexual maturation of adult worms, a systemic hypersensitivity reaction occurs in the host, called Katayama syndrome (for *S. japonicum*) or the toxaemic form (for *S. mansoni*), which is associated with an intense Th1 response (Neves, 1992; Ross *et al*., 2007; Caldas *et al*., 2008; Langenberg *et al*., 2019). During primary infections in non-immune individuals, the main symptoms related to this systemic inflammation include a high fever accompanied by chills, profuse sweating, asthenia, myalgia, headache and a non-productive cough (Schwartz *et al*., 2000; Bottieau *et al*., 2006).

After parasite oviposition (between 5th and 6th weeks post-infection), there is a change in the profile of immune mediators produced by the host, and the immune response becomes predominantly Th2, which is associated with increasing production of IL-4, IL-5, IL-9 and IL-13 (MacDonald *et al*., 2002; Pearce *et al*., 2004; Bartley *et al*., 2006; Burke *et al*., 2009). Such changes are responses to soluble egg antigens (SEAs) (Hams *et al*., 2013), that is composed of a complex mixture of immunostimulatory antigens that are known for their ability to condition DCs to initiate the induction of a Th2 profile (Mouser *et al*., 2019).

DCs detect, capture and process antigens derived from eggs of *S. mansoni* (Cervi *et al*., 2004; van Liempt *et al*., 2007), resulting in their ability to lead to Th2 polarization both *in vitro* and *in vivo* (de Jong *et al*., 2002; MacDonald *et al*., 2002; Perona-Wright *et al*., 2006). The main antigens responsible for this potent induction of a Th2 response are glycoproteins omega 1 (*ω*-1) and IPSE (IL-4-inducing principle of *S. mansoni* eggs)/alpha 1 (*α*-1) (Schramm *et al*., 2006; Meevissen *et al*., 2010). Glycoprotein *ω*-1 is present in both SEAs (Dunne *et al*., 1991) and E/S products from live eggs (Cass *et al*., 2007), and activates DCs (via C-type and Toll-type lectin receptors), which in turn promotes Th2 differentiation, the main source of type 2 cytokines such as IL-4, IL-5 and IL-13 (Everts *et al*., 2009). On the other hand, a previous study (Schramm *et al*., 2006) showed that the glycoprotein IPSE/*α*-1 is exclusively released from mature eggs, but likely possesses the same potential to initiate a Th2 response during *S. mansoni* infection. IPSE/*α*-1 binds to immunoglobulin and activates basophils, leading to the release of histamine and facilitating the production of Th2-type cytokines, mainly IL-4 and IL-13 (Schramm *et al*., 2007; Meyer *et al*., 2015; Knuhr *et al*., 2018). Thus, the Th2 response (Fig. 2) is related to low production of IFN-*γ* and high concentrations of anti-inflammatory cytokines (IL-4, IL-5, IL-10 and IL-13) (Grzych *et al*., 1991; Pearce *et al*., 2004; Zheng *et al*., 2020).

## Mechanisms associated with macrophage polarization

Macrophages are cells of the innate immune system that have phagocytic capacity and are involved in the elimination of foreign particles from the body (Gordon and Martinez-Pomares, 2017; Uribe-Querol and Rosales, 2020) and in the presentation of antigens, constituting an important link between innate and adaptive immunity. These cells are part of the mononuclear phagocytic system and are implicated in tissue homoeostasis and various infectious and inflammatory processes (Rahman *et al*., 2017; Shapouri-Moghaddam *et al*., 2018).

**Fig. 3.**
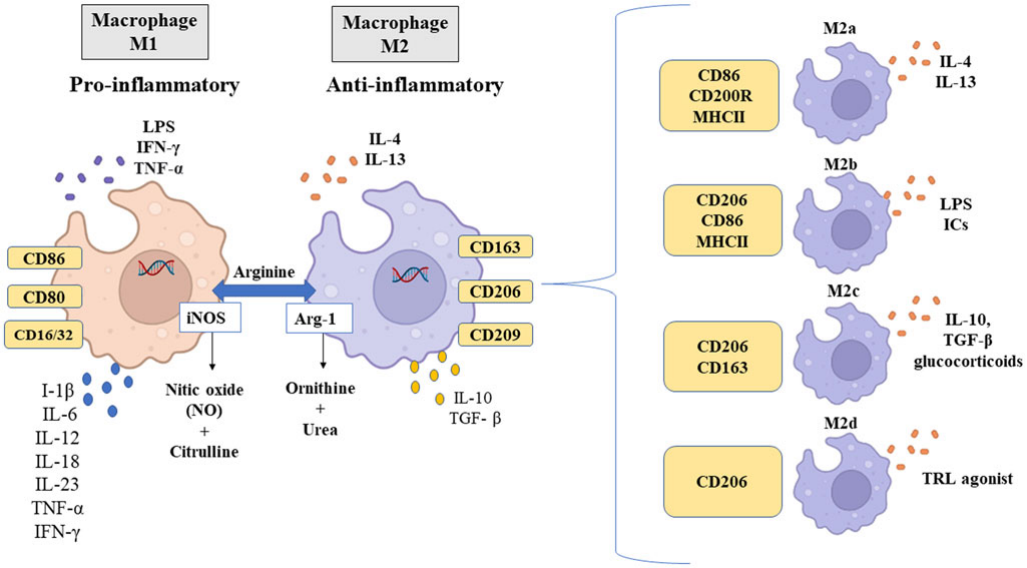
Different macrophage phenotypes, specific stimuli and markers. *Source*: Created with BioRender.com.

Macrophages are activated during phagocytosis or by contact with molecular patterns associated with pathogenic microorganisms. This activation results in inflammatory responses and increased production of cytokines and/or physicochemical factors and, consequently, can differentiate into various phenotypes depending on the state and changes in the microenvironment (Schmall *et al*., 2015; Murray, 2017). There are 2 main subtypes of macrophages classified according to the expression of their cell surface markers, production of specific factors and biological activities: classically activated or inflammatory M1 macrophages and alternatively activated or anti-inflammatory M2 macrophages (Parisi *et al*., 2018) (Fig. 3). Macrophage subtypes play a role in the initiation and/or progression of many diseases. The M1/M2 paradigm emerged as homologous with the one previously described for Th response profiles, which also presents 2 subtypes: Th cell type 1 (Th1) and type 2 (Th2) (Mills, 2015).

M1 macrophage subtypes polarize in the presence of Th1 cytokines such as IFN-*γ* and TNF-*α* or when exposed to inflammatory molecules such as lipopolysaccharides (LPS) (Yunna *et al*., 2020), through the following mechanisms: (1) JAK/STAT (Janus kinase/signal transducer and transcriptional activator) signalling pathway. IFN-*γ* activates JAK-inducing phosphorylation of STAT1, which in turn leads to macrophage polarization to M1 (Wang *et al*., 2020); (2) Toll-like receptor (TLR) 4/nuclear factor *κ*B (NF-*κ*B) signalling pathway. LPS binds to TLR4 to activate NF-*κ*B and activator protein 1 (AP-1), promoting the expression of inflammatory factors (Chen *et al*., 2017*b*; Ciesielska *et al*., 2021) and (3) cytokine signalling through specific receptors that activate AP-1 (Liu *et al*., 2014).

M1 macrophages are recruited soon after lesion formation and are mainly involved in the initial response to infectious processes (Vannella and Wynn, 2017). These increase local inflammation, producing large amounts of pro-inflammatory cytokines, including IL-1*β*, IL-6, IL-12, IL-18, IL-23, TNF-*α* and IFN type 1 (Shapouri-Moghaddam *et al*., 2018), as shown in Fig. 3. The M1 macrophage phenotype expresses high levels of inducible nitric oxide synthase (iNOS), major histocompatibility complex class II (MHC II), CD16/32, CD80 and CD86, as well as chemokines that attract Th1 cells, including CXCL9 and CXCL12 (Orecchioni *et al*., 2019). Functionally, M1 macrophages are characterized by antimicrobial and antitumour activities and participate in the elimination of infectious agents through the activation of nicotinamide adenine dinucleotide phosphate (NADPH) oxidase and, consequently, the generation of reactive oxygen species (ROS) (Murray *et al*., 2014).

On the other hand, M2 macrophages are induced by Th2 cytokines IL-4 and IL-13 (Fig. 3), mainly *via* STAT6 activation (Sica and Mantovani, 2012; Enderlin *et al*., 2014). This pathway is extremely important, as IL-4 inhibits M1 and induces M2 polarization (He *et al*., 2020). Gao *et al*. (2015) demonstrated that the expression of STAT6 was positively regulated by curcumin and by the secretion of IL-4 and IL-13, capable of inducing M0 and M1 macrophages to polarize into M2. IL-4 type I and type II receptors also activate STAT6 (Gong *et al*., 2017), which in turn induces the transcription of typical M2 polarization genes, such as mannose receptor 1, type *α* resistin (Retnla) and chitinase 3-like 3 (Chi3l3, Ym1) (Martinez and Gordon, 2014). M2 polarization can also be induced by IL-10 through STAT3 activation (Yin *et al*., 2018). However, the STAT6 pathway is considered to activate M2 macrophages (Murray, 2017).

The M2 macrophage phenotype has a profile of anti-inflammatory cytokines, characterized by low production of IL-1, IL-6 and TNF-*α*, and high production of IL-10 and transforming growth factor-beta (TGF-*β*) (Fig. 3), as well as chemokines CCL1, CCL17, CCL18, CCL22 and CCL24 (Yunna *et al*., 2020). Additionally, this phenotype can be characterized by the expression of arginase 1 (Arg-1), CD163, CD209 and CD206. CD206 interacts with glycoproteins and glycolipids found on the surfaces of pathogens (Suzuki *et al*., 2018; Xu *et al*., 2019). Thus, CD206 plays a role in immunological recognition of pathogens after antigen internalization and presentation (Hussell and Bell, 2014). Functionally, M2 macrophages can inhibit inflammation, promote tissue repair and wound healing, prevent parasitic infection and have proangiogenic and profibrotic properties (Jetten *et al*., 2014; Braga *et al*., 2015). Furthermore, because M2 macrophages produce complex cytokines and are characterized by the functional expression of alternative activation markers, they can be divided into 4 subtypes: M2a, M2b, M2c and M2d (Yao *et al*., 2019). These subtypes differ from each other based on their cell surface markers, secreted cytokines and biological functions, as is demonstrated in Fig. 3.

M2a macrophages are induced by the cytokine IL-4 or IL-13 and express high levels of CD86, CD200R and MHC II and low levels of CD14 and TLR4 (Yao *et al*., 2019). In addition to being major producers of CCL24, CCL17 and CCL22, they use CCR3 and CCR4 receptors, resulting in the recruitment of eosinophils, basophils and Th2 cells, promoting the upregulation of a type 2 immune response (Fraternale *et al*., 2015). M2b-type macrophages are induced by immune complexes, LPS or IL-1 receptor antagonist and are characterized by increased expression of CD206 and CD86 (Viola *et al*., 2019). Upon activation, this subtype secretes pro- and anti-inflammatory cytokines TNF-*α*, IL-1*β*, IL-6 and IL-10 and functions in regulating the immune response and inflammation (Wang *et al*., 2019). M2c macrophages are induced by IL-10, TGF-*β* or glucocorticoids, and express CD206 and CD163, in addition to secreting IL-10, TGF-*β*, CCL16 and CCL18, which play crucial roles in the phagocytosis of apoptotic cells (Ross *et al*., 2021). Finally, induced by TLR antagonists, M2d macrophages express high levels of CD206, IL-10 and iNOS, secrete CCL5, CXCL10 and CXCL6 and express low levels of IL-12 and TNF-*α* (Viola *et al*., 2019). This subtype also secretes the vascular endothelial growth factor and promotes angiogenesis and tumour progression (Ferrante *et al*., 2013). Notably, all subtypes of M2 macrophages express IL-10.

M1 and M2 macrophages can also be differentiated by the way they metabolize arginine, as shown in Fig. 3. M1 macrophages metabolize arginine by the enzyme iNOS to produce nitric oxide (NO) and citrulline; on the contrary, M2 macrophages metabolize arginine by Arg-1 to produce l-ornithine and urea, a precursor molecule of polyamines involved in tissue repair and cell proliferation (Rath *et al*., 2014; Yang and Ming, 2014).

The 2 macrophage populations must be balanced to maintain homoeostasis and to protect the organism. Once an imbalance occurs, the exacerbated activity of M1 or M2 macrophages can lead to the development of inflammatory diseases or host immunosuppression (Sica *et al*., 2015). However, the remarkable plasticity of macrophages confers significant benefits to the host, especially in the course of chronic helminth infections (Lechner *et al*., 2021) since it limits excessive tissue damage when it is unable to overcome the initial injury. This feature has been well-documented in schistosomiasis.

## Participation of M1 and M2 macrophages in the response to *Schistosoma* infection

Initially, blood monocytes differentiate into macrophages at inflammatory sites (Rückerl and Cook, 2019) and exhibit high plasticity as a result of exposure to various stimuli, signalling molecules, nutrients and metabolites in the context of schistosomiasis (Cortes-Selva and Fairfax, 2021). These phagocytes can exert pro-inflammatory or anti-inflammatory functions (Zhu *et al*., 2014) in different clinical forms of schistosomiasis (acute and chronic phases) (Fig. 2). In the acute phase, macrophages secrete pro-inflammatory cytokines and consequently increase inflammation, recruit more immune cells and promote the formation of the initial granuloma. In the chronic phase, macrophages have an immunoregulatory activity to decrease the damage caused by granulomas (Wolde *et al*., 2020).

During the life cycle of *S. mansoni* and *S. japonicum*, several antigens are excreted by their different evolutive forms (Curwen *et al*., 2006; Jang-Lee *et al*., 2007; Acharya *et al*., 2021). For example, Sm16 – a low molecular weight protein that is secreted by *S. mansoni* cercariae, helps the parasite to enter the host's skin (Brännström *et al*., 2009; Sanin and Mountford, 2015). Sm29, present in the tegument of schistosomula and adult *S. mansoni* worms, can induce the maturation and activation of human monocyte-derived DCs (Cardoso *et al*., 2008; Lopes *et al*., 2019). Sj-C is an example of a protein secreted from the tegument of *S. japonicum*, which may suppress the presentation of exogenous antigens by DCs (He *et al*., 2011; Chen *et al*., 2017*a*). IPSE/*α*-1 and *ω*-1 are examples of proteins secreted by *S. mansoni* eggs, which help direct a Th2 response (Everts *et al*., 2009; Knuhr *et al*., 2018). These molecules can induce the activation and modulation of innate and adaptive immune responses and facilitate the evasion of the parasite from the host-defense mechanisms (Jenkins *et al*., 2005*a*, 2005*b*; Hai *et al*., 2014; Hambrook and Hanington, 2021). *Schistosoma* antigens can be proteins (such enzymes), polysaccharides and the most commonly used are crude extracts prepared by breaking up worms, larvae or eggs (Doenhoff *et al*., 1993; Doenhoff, 1998). Thus, it is clear that antigen changes in the microenvironment during schistosomiasis are important for the polarization of macrophages to the M1 or M2 profile (Xu *et al*., 2014; Sanin and Mountford, 2015; Assunção *et al*., 2017).

According to Tables 1 and 2, we highlight some *in vitro* and *in vivo* studies that demonstrate the relationship between the stimulation of *S. japonicum* and *S. mansoni* antigens and macrophage polarization. In addition, we also highlight other molecules involved in macrophage polarization in schistosomiasis, providing molecular evidence of great relevance in the process of differentiation of these cells, which will be discussed in this article.

**Table 1.** Molecules and/or antigens involved in macrophage polarization in *Schistosoma mansoni* infection

Macrophage profile	Molecule/antigen	Experimental model	Type of study	References
M1	Schistosomules	C57BL/6 mice	*In vivo*	Menson and Wilson (1990)
M2	SEA	BMDCs and DCs from C57BL/6 or TLR4 mice	*In vitro*	Goh *et al*. (2009)
		Macrophages derived from human monocytes		
M2	p16^INK4a^	Macrophages derived from mouse bone marrow	*In vitro*	Cudejko *et al*. (2011)
		Chimaeric mice	*In vivo*	
M2	Cercariae	Peritoneal macrophages	*In vitro*	Vanella *et al*. (2014)
		Mice	*In vivo*	
		IL4R*α*^flox/Δ^ LysM^WT/Cre^		
	SEA	Mutant mice		
		*LysM*^Cre/+^ *Shp2*^flox/flox^ (control) and *LysM*^Cre/+^: *Shp2*^flox/flox^ (*Shp2*^Δ/Δ^)		
M2	CD14 TLR co-receptor	Mice *Cd14*^−/−^	*In vivo*	Tundup *et al*. (2014)
		Wild-type mice (wt)		
M1 profile lock	Sm16 antigen	Macrophages derived from mouse bone marrow	*In vitro*	Sanin and Mountford (2015)
M2	LPC	Peritoneal macrophages and bone marrow derivatives of mice	*In vitro*	Assunção *et al*. (2017)
M2	IPSE/*α*-1	Human peripheral blood cells (monocytes and basophils)	*In vitro*	Knuhr *et al*. (2018)

**Table 2.** Molecules and/or antigens involved in macrophage polarization in *Schistosoma japonicum* infection

Macrophage profile	Molecule/antigen	Experimental model	Type of study	References
M1/M2	NCA	Macrophages RAW264.7	*In vitro*	Xu *et al*. (2014)
	ACA			
	SWAP	Mice	*In vivo*	
	SEA	C57BL/6J		
M1/M2	SWA	Peritoneal macrophages of mice	*In vitro*	Zhu *et al*. (2014)
	SEA			
M1	EVs	Macrophages RAW264.7	*In vitro*	Wang *et al*. (2015)
M2	Corilagin	Ana-1 cell line	*In vitro*	Li *et al*. (2017)
		C57BL/6 mice	*In vivo*	
M2b	Egg-derived ES antigens	Macrophages derived from bone marrow of wild-type mice and TLR2^−/−^	*In vitro*	Gong *et al*. (2018)
		C57BL/6 mice	*In vivo*	
M2	Sj16	Mouse peritoneal macrophages	*In vitro*	Shen *et al*. (2019)
		BALB/c mice	*In vivo*	
M1	EV miRNAs EVs	Macrophages RAW264.7	*In vitro*	Liu *et al*. (2019)
		BALB/c mice and rabbits	*In vivo*	
M2	Eggs	Mouse peritoneal macrophages	*In vitro*	Ye *et al*. (2020)
		Mice	*In vivo*	
		Kunming		
M1	SWA	Macrophages RAW264.7	*In vitro*	Shen *et al*. (2021)
		C57BL/6 mice	*In vivo*	
M2	SEA	Macrophages J774A.1	*In vitro*	Yu *et al*. (2021)

## Cercariae and schistosomula antigens can induce an M1 profile

During the penetrating of the human skin, cercariae of *S. mansoni* and *S. japonicum* release E/S products, which have remodelling and immunoregulatory functions (Liu *et al*., 2015; Sanin and Mountford, 2015), that facilitate their penetration and subsequent establishment in the host's body, in the form of schistosomula (Janssen *et al*., 2016). This phase represents the first contact with innate immune responses in the skin, especially Langerhans cells, which are considered tissue-resident macrophages (West and Bennett, 2018). These cells phagocytize E/S and secrete pro-inflammatory (IL-6 and IL-12p40) and anti-inflammatory (IL-10) cytokines in a TLR-dependent manner (Jenkins *et al*., 2005*a*, 2005*b*).

One of the ways in which macrophages are activated is through the action of TLRs. These receptors are a family of pattern recognition receptors that are important for innate immune response (El-Zayat *et al*., 2019). These receptors recognize invading pathogens, trigger innate immune responses and subsequently initiate adaptive immunity against infections, including Gram-positive and Gram-negative bacteria, fungi, viruses and parasites (Lu *et al*., 2018). These receptors mediate macrophage recognition by microbial ligands, inducing the expression of microbicidal molecules and cytokines *via* the adapter protein MyD88 (Jin *et al*., 2019). Xu *et al*. (2014) showed that normal cercariae antigen (NCA) and attenuated cercariae antigen (ACA) from *S. japonicum* induced polarization to the M1 profile, with increased levels of IL-12, CD136/32 and iNOS (Table 2). However, these values decreased when the TLR4 pathway blockers were used. Thus, the authors suggested that the polarization of the M1 profile is dependent on the TLR4 pathway and this may play a protective role in *S. japonicum* infection (Tang *et al*., 2021).

In fact, the TLR4 pathway is extremely important for the polarization of macrophages to the M1 phenotype, as demonstrated in some studies (Freitas *et al*., 2016; Shi *et al*., 2020). Sanin and Mountford (2015) demonstrated that Sm16 (a molecule produced by *S. mansoni* cercariae) is able to block TLR4 and TLR3 pathways in human monocyte, which negatively affect the classic activation of macrophages (M1) in response to IFN-*γ* (Table 1). This is considered an important mechanism of immune evasion promoted by *S. mansoni* because it limits the production of NO, which is toxic to the parasite (Shiels *et al*., 2020).

After complete transformation from a cercariae into a schistosomula, the larva migrates into the bloodstream, travelling through the lungs until reaching maturation in the mesenteric veins. This stage of the cycle is also characterized as a key target for the elimination of infection through innate host immune responses (Houlder *et al*., 2021). Some histological studies conducted in the lungs of mice infected with *S. mansoni* and *S. japonicum* showed inflammatory foci consisting of neutrophils, eosinophils and macrophages (Crabtree and Wilson, 1986; Burke *et al*., 2011).

Macrophages function as cytotoxic cells, mainly in schistosomula (James and Glaven, 1989). Oswald *et al*. (1994) demonstrated that macrophages could produce NO, leading to schistosomula death in animal models independent of the production of pro-inflammatory cytokines. In contrast, Cardoso *et al*. (2008) determined that the antigen Sm29, present in the integument of *S. mansoni* schistosomula, induced a Th1-type immune response, with an increase in pro-inflammatory cytokines (IFN-*γ*, TNF and IL-12) in mice, leading to a reduction in worm burden and liver pathology. James *et al*. (1998) demonstrated that IFN-*γ* was a cytokine of great importance in the activation of macrophages in the lungs for the immunological killing of *S. mansoni* larvae and played a critical role in protective immunity.

In the early stages of schistosomiasis, lung macrophages may have an M1 phenotypic trait. Menson and Wilson (1990) characterized the expression of surface markers in alveolar macrophages associated with the immune response to *S. mansoni*. The authors demonstrated an increase in IFN-*γ* expression in the lungs of C57BL/6 mice and suggested that activated macrophages might be responsible for initiating and maintaining focal inflammation that blocks parasite migration (Table 1).

## Worm antigens can induce an M1 or M2 profile

During the acute phase of *Schistosoma* infection, before parasite oviposition (approximately 5–7 weeks post-infection), immune responses are largely of the CD4+ Th1 type, associated with increased numbers of M1 macrophages that produce IL-12, IL-6, TNF-*α* and NO (Pearce *et al*., 1991; Coulson *et al*., 1998; Gordon, 2003). These early pro-inflammatory responses are mainly related to the antigens from immature worms (schistosomula) during their migration (Wilson, 1998; Egesa *et al*., 2018). Activation of these responses may be through binding to TLR and C-type lectin receptors on macrophages; however, further studies are needed to clarify this mechanism of macrophage activation *via* schistosomula antigens.

In contrast to the schistosomula antigens, the adult worm antigen preparations [soluble worm antigen (SWAP or SWA)] were better explored in experimental studies. Although the antigenic composition is not the same as the live worm, the use of SWAP or SWA constitutes a valuable experimental tool to evaluate many aspects of immune responses promoted by different host cells (Xu *et al*., 2014; Zhu *et al*., 2014). This antigen is the easiest to obtain and is essentially an extract based on Tris-HCl or phosphate-buffered saline from mixed male and female worms and prepared in various ways, either by homogenization, sonication or freeze/thaw (or a combination of these) (Grenfell *et al*., 2012; Neves *et al*., 2015). Some studies have demonstrated that SWAP could induce an M1-like profile (Xu *et al*., 2014; Zhu *et al*., 2014). Thus, Zhu *et al*. (2014), when performing a co-culture of peritoneal macrophages obtained from mice with *S. japonicum* SWA (Table 2), observed that there was an increase in the expression of specific markers related to M1 (TNF-*α*, IL-12, CXCL9, CXCL10, CXCL11 and iNOS).

Aiming to understand which mechanisms lead SWAP to induce polarization of the M1 profile, Shen *et al*. (2021) demonstrated that this antigen promoted the expression of a protein called lipocalin 2 (LCN2) and, consequently, induced the M1 profile of macrophages (Table 2) through the upregulation of the NF-*κ*B signalling pathway. It has already been reported that this protein is increased in macrophages and can potentiate the M1 phenotype of microglia in the central nervous system (Jang *et al*., 2013). The NF-*κ*B signalling pathway can activate macrophages to produce M1 polarization upon LPS induction (Liu *et al*., 2017). In addition, some studies have shown that this pathway could regulate the expression of LCN2, thereby stimulating the inflammatory response in infectious processes (Zhao and Stephens, 2014; Ghosh *et al*., 2017).

In addition to the antigens of adult worms that induce an M1 profile, studies have shown that adult worms of *S. mansoni* and *S. japonicum* also release extracellular vesicles (EVs), known as exosomes, which modulate the host immune response (Nowacki *et al*., 2015; Wang *et al*., 2015; Sotillo *et al*., 2016; Zhu *et al*., 2016). Exosomes are membrane-bound vesicles secreted by various types of mammalian cells in normal and diseased states (Avni and Avni, 2021). Exosomes play an important role in cell–cell communication and have been implicated in the regulation of cell development, immune regulation, angiogenesis and cell migration (Raposo and Stoorvogel, 2013; Zhu *et al*., 2016). Wang *et al*. (2015) observed that RAW264.7 macrophages, when cultured with exosome-like vesicles isolated from *S. japonicum*, exhibited an M1 profile (Table 2), due to the increase in the surface markers CD16/32, iNOS and TNF-*α*. Liu *et al*. (2019) investigated miRNAs from *S. japonicum* EVs and found that they increased macrophage proliferation *in vitro* (RAW264.7) and *in vivo* (mice and rabbits) as well as TNF-*α* expression. miRNAs are involved in the regulation of the development, differentiation and activation of immune cells, including macrophages (Montagner *et al*., 2014; Mehta and Baltimore, 2016). Thus, the polarization of M1 induced by schistosome EVs may represent an important mechanism for parasite survival in vertebrate hosts, *via* modulation of the immune response. However, there are still controversies about the possible role of the schistosome tegument as a source of EVs, because, to date, no study has been performed to prove the exact origin of these vesicles (Wilson and Jones, 2021).

On the other hand, adult worm products can also bias the M2 profile (Smith *et al*., 2018). Indeed, Xu *et al*. (2014) showed that adult *S. japonicum* worms could induce an M2 macrophage profile. The authors, when stimulating RAW264.7 macrophages with SWAP from *S. japonicum*, observed an increase in the expression of surface markers (CD16/32 and CD206) and in the production of cytokines (IL-12 and IL-10), suggesting that this antigen could induce both M1 and M2 macrophage profiles. The potential explanation for this could be related to how the antigens of adult worms were obtained since some adult female worms possess eggs in the process of maturation into their uterus/ootype, and consequently, this antigen could have been contaminated with SEAs. However, further studies are needed to understand macrophage polarization by SWAP and its relationship with SEA contamination.

Besides the classical macrophage polarization (M1 and M2), products excreted by schistosomes, such as haemozoin, are also able to induce immunomodulation. Adult worms of *S. mansoni* acquire nutrients by haematophagy of the host's blood, and this process can form toxic haem for the parasite (Zussman *et al*., 1970). However, the schistosomes are able to neutralize the free haem in their intestine through crystallization in haemozoin (Oliveira *et al*., 2000). This haemozoin is regurgitated by the worms into the host bloodstream and can be accumulated in the liver (Kloetzel and Lewert, 1966), which may activate the immune response of the host. From this perspective, a previous study (Truscott *et al*., 2013) highlighted that haemozoin formed from *S. mansoni* is able to maintain the M2 macrophage profile previously activated by IL-4 stimulation, but also exerts specific modulatory effects on these cells (Table 1). These authors showed that haemozoin mediated the suppression of *Retnla* (resistin-like molecule-*α* or *Fizz1*) expression and Retnla protein secretion in the M2 macrophages. The role of Retnla during experimental schistosomiasis is associated with the limitation of Th2 inflammatory response (Pesce *et al*., 2009). However, further studies are necessary to better explain the possible impact of haemozoin in the immunopathology of schistosomiasis.

## SEAs can induce an M2 profile

After maturation of the adult worms and subsequent oviposition, the activation of a type 2 profile begins in response to the soluble antigens secreted by the eggs of *S. mansoni* and *S. japonicum* (Tables 1 and 2) (Cheever *et al*., 2000; Pearce and MacDonald, 2002; Pearce *et al*., 2004; Burke *et al*., 2009; Costain et al., 2018). The type 2 profile of schistosomiasis is characterized by the expansion of Th2 cells, eosinophils and basophils, and increased production of IL-4, IL-5 and IL-13 (Hams *et al*., 2013; Schwartz *et al*., 2014), as previously described. IL-4 and IL-13 protect hosts against various helminth parasites by signalling through the IL-4R*α* chain (Barron and Wynn, 2011; Jenkins *et al*., 2011). The production of these cytokines reduces the inflammation levels produced by the type 1 profile of the initial stage of acute phase, preventing acute pathology, such as intestinal haemorrhage and liver damage; however, the Th2 immune response is responsible for the formation of hepatic and intestinal granulomas (Brunet *et al*., 1997; Hams *et al*., 2013; Zheng *et al*., 2020).

Granulomas are essential for sequestering toxic antigens produced by eggs and preventing further tissue damage. However, if unregulated by the immune response of the host, granulomas grow excessively and progress to fibrotic stages, which are responsible for severe forms of the disease, such as cirrhosis, portal hypertension, liver failure and even host death (Lenzi *et al*., 1998; Cheever *et al*., 2000; Takaki *et al*., 2021). Macrophages are one of the main cellular components of hepatic granulomas (Beljaars *et al*., 2014; Schwartz and Fallon, 2018). Recent studies have demonstrated that M2 macrophages play a direct and critical role in fibrosis, granuloma maintenance, tissue repair and host survival (Cortes-Selva *et al*., 2018; Song *et al*., 2020). Ye *et al*. (2020) showed that M2 macrophage markers (CD200R, Arg-1 and Ym1) were increased in the liver, spleen, large intestine and peritoneal macrophages of *S. japonicum*-infected mice. Jenkins *et al*. (2011) observed that IL-4/IL-13 signalling *via* IL-4R*α* induces an alternative phenotype in resident macrophages. In this sense, a study performed with macrophages derived from the bone marrow of mice infected with *S. mansoni* demonstrated that the tumour suppressor gene p16 ^INK4a^ was an excellent modulator of the activation and polarization of macrophages induced by IL-4 through the JAK2–STAT1 pathway (Cudejko *et al*., 2011).

Egg antigens induce granulomas, consisting mainly of M2 macrophages (Yu *et al*., 2021). Zhu *et al*. (2014) showed that peritoneal macrophages obtained from healthy mice, when stimulated with *S. japonicum* SEAs, expressed high levels of chemokines (CCL2, CCL17 and CCL22), IL-10 and Arg-1. Similarly, Xu *et al*. (2014), after stimulating RAW264.7 macrophages with *S. japonicum* SEAs, also observed higher levels of IL-10. In chronic schistosomiasis infection, the main function of IL-10 is to control liver damage and regulate antifibrotic processes (Dewals *et al*., 2010; Kamdem *et al*., 2018). Previous studies have shown that low levels of IL-10 expression are related to liver fibrosis in *S. mansoni*-infected patients (Mutengo *et al*., 2018). On the other hand, little is known about the mechanisms by which an SEA preferentially induces M2 macrophage differentiation. Previous studies have demonstrated that an SEA from *S. mansoni* could induce the expression of the notch Jagged1 ligand in mice and human macrophages, suggesting that Jagged1 might have a specific role in the M2 polarization process of macrophages (Goh *et al*., 2009) (Table 1). Macrophages found in liver tissues exhibit functional M2 polarization, which is dependent on the activation of notch1/Jagged1 signalling (Zheng *et al*., 2016).

During *S. mansoni* infection, basophils detect egg IPSE/*α*-1 glycoprotein and stimulate the production of IL-4 and IL-13, which trigger the alternative activation of human monocytes (Table 1), as observed by the increased expression of CD206 and CD209 (Knuhr *et al*., 2018). IL-13 is a key cytokine that induces M2 macrophage polarization *via* the IL-13*α*1 signalling pathway (Chiaramonte *et al*., 1999; Liu *et al*., 2012). Li *et al*. (2017) performed a study with corilagin, an active component of many medicinal plants, and found that this component could suppress *Schistosoma* egg-induced liver fibrosis by inhibiting M2 macrophage polarization (Table 1) in the IL-13R*α*1 signalling pathway. Corilagin has great potential to reduce liver fibrosis caused by egg antigens in *S. japonicum* infection by decreasing the expression of molecules associated with the IL-13/STAT6 signalling pathway in liver M2 macrophages (Du *et al*., 2016).

Signaling *via* TLR2 may be another way egg antigens polarize M2 macrophages during schistosomiasis. Gong *et al*. (2018) showed that antigens derived from *S. japonicum* eggs could activate macrophages, which exhibit M2b polarization dependent on NF-*κ*B signalling, mediated by the MyD88/mitogen-activated protein kinase (MAPK) pathway in a TLR2-dependent manner (Table 1). In contrast, Tundup *et al*. (2014) showed that the CD14 TLR co-receptor was upregulated in hepatic macrophages after *S. mansoni* infection and acted as a crucial negative regulator of M2 polarization, possibly as part of a parasitic defense mechanism against granuloma formation (Table 1). Gao *et al*. (2013) observed that an SEA of *S. japonicum*, known as SjEA, upregulated programmed death ligand 2 (PD-L2) expression in mouse bone marrow-derived macrophages (BMDCs) via TLR2, which binds PD-1 primarily on CD4+ T cells. This mechanism can help inhibit the T cell response during *S. japonicum* infection.

Lysophosphatidylcholine (LPC) from *S. mansoni* eggs can also induce macrophage differentiation into the M2 phenotype (Assunção *et al*., 2017), as shown in Table 1. The authors observed that LPC from *S. mansoni* activates peroxisome proliferator-activated receptor gamma (PPAR-*γ*), a transcription factor necessary for M2 polarization, leading to higher expression of Arg-1 and CD206, while increasing the production of IL-10, TGF-*β* and PGE2 in peritoneal macrophages *in vitro*. *Schistosoma mansoni* eggs induced a 7-fold increase in PPAR-*γ* expression in human liver cell cultures (Anthony *et al*., 2010). PPAR-*γ*, in addition to being of great importance in M2 polarization, can regulate lipid uptake and metabolism (Ahmadian *et al*., 2013; Abdalla *et al*., 2020).

Fang *et al*. (2015) showed that BMDCs from C57BL/6 mice, when stimulated with a specific *S. japonicum* egg protein known as SjE16.7, promoted the production of pro- (IL-12, IL-6 and TNF-*α*) and anti-inflammatory (IL-10) cytokines through the phosphorylation of MAPKs and increased the expression of MHC II on the surface of macrophages. Previous studies have shown that *S. mansoni* and *S. japonicum* egg antigens could stimulate the MAPK pathway in macrophages (Wang *et al*., 2006; de Andrade *et al*., 2014). MAPKs are essential transmitters of extracellular signals that can mediate key cellular processes, including cell differentiation, division and death (Yang *et al*., 2003). Thus, SjE16.7 is a potent macrophage activator. However, in another study, Shen *et al*. (2019), when using the Sj16 antigen, noticed that it decreased hepatic granulomas in mice infected with *S. japonicum* and associated this improvement with the suppression of cytokine production, such as IFN-*γ*, TNF-*α*, IL-4 and IL- 6. The authors reported that the mechanisms of Sj16 attenuation of hepatic granulomatous inflammation and fibrosis in these infected mice might be related to the induction of macrophages for M2 polarization (Table 2). These authors also demonstrated, by flow cytometry, the increase in the expression of CD206 after stimulation of Sj16 in peritoneal macrophages and leucocytes from the livers of mice. Corroborating these results, Hu *et al*. (2009) showed that Sj16 decreased the levels of pro-inflammatory cytokines, such as IL-6 and TNF-*α*, and increased the levels of IL-10 in RAW264.7 macrophages. Vannella *et al*. (2014) observed that mice infected with *S. mansoni* showed an increase in M2 macrophages that expressed Arg-1, which attenuated the progression of inflammation and fibrosis (Table 1). Stimulation of RAW264.7 macrophages with another *S. japonicum* egg protein (SjCP1412) also increased the expression of CD206, Arg-1 and IL-10, which are related to M2-type macrophage differentiation (Ke *et al*., 2017). Overall, these findings emphasize that M2 macrophages are important in reducing the lesions caused by schistosomiasis through downregulation of the Th1 response and inflammation promoted by egg antigens. Additionally, the role of these cells was previously investigated in a mouse model of liver injury induced by acetaminophen (paracetamol) (Starkey Lewis *et al*., 2020). The authors demonstrated that the injection of M2 macrophages in this experimental model was able to rapidly reduce liver damage and inflammation. These data indicate that M2 macrophages may constitute a new potential cell-based therapy for this disease. Based on this, it seems promissory also to apply these cells in the immunotherapy of schistosomiasis.

Interestingly, despite being produced by M1 macrophages, a recent study demonstrated that the production of ROS by egg antigens may be a potential mechanism for M2 macrophage differentiation (Table 2). ROS have several biological activities, such as participation in innate and adaptive immune responses, and can be cytotoxic against pathogens (Canton *et al*., 2021). Yu *et al*. (2021) observed that a significant increase in ROS in the liver of mice infected with *S. japonicum* was related to fibrosis and the differentiation of M2 macrophages. The authors hypothesized that their findings were due to NADPH oxidase (NOX2) inhibiting SEA-stimulated ROS production in macrophages, suggesting that NOX might act as the main source of ROS production in SEA-stimulated macrophages. NADPH oxidase is the first source of ROS identified in macrophages (Nathan *et al*., 2013). Macrophages produce large amounts of ROS, primarily through NOX2 activation (Paik *et al*., 2014). Thus, the production of ROS induced by schistosome eggs may be a target for the treatment of schistosomiasis.

## Future perspectives and final considerations

Findings about the mechanisms behind macrophage activation during different metabolic profiles in human diseases present an exciting prospect, as there are pathologies that have been associated with a particular macrophage phenotype. In this context, the polarization of macrophages in schistosomiasis and their consequent ability to promote an effective immune response seem to be an attractive therapeutic approach associated with conventional chemotherapy treatments.

Overall, the findings highlighted in this review demonstrate the relevance and complexity of understanding the mechanisms involved in macrophage polarization (M1/M2) in schistosomiasis. The *S. japonicum* and *S. mansoni* antigens in macrophage polarization are particularly important in this process. These products have been shown to have immunomodulatory effects in different phases of schistosomiasis and are seen as potential therapeutic targets for this disease, especially in the chronic phase. Among the potential therapeutics, the combination of different schistosome antigens can result in higher levels of host protection, stimulating an adequate immune response for either an M1 or M2 profile; however, this can only be achieved after many *in vitro* and *in vivo* experiments.
